# Foreign Summary

**Published:** 1840-05-23

**Authors:** 


### FOREIGN SUMMARY.

*Inhalation in Tubercular Phthisis Pulmonalis.* By Sir Charles Scudamore.—As the practical consideration of any new remedy which may be proposed for the relief of disease, must be one of the useful purposes of a periodical journal, I trust that some further observations on the question of Inhalation in Tubercular Phthisis Pulmonalis, and in Chronic Bronchitis, may be offered to you as an acceptable contribution.

It was in 1830 that I published the first edition of my Cases in illustration of the subject; and therefore I may lay claim to a very extended experience of this particular method of treatment, and to the consequent opportunity of forming a more mature judgment of its merits. But I wish, in the first place, to be understood as speaking of this remedy in the light of an auxiliary, and one not exclusive of other treatment. It is to be considered that every practitioner, in consenting to adopt this treatment, may at the same time employ those general means in which he is accustomed to confide. I believe that I have derived from this method of practice more success, and a larger share of satisfactory result, even where cure was not attainable, than the majority who have adopted it. This I can only explain from my greater study of the action of remedies administered in the way of inhalation; from my greater confidence in their power and usefulness; and consequently a more patient perseverance in their employment. Yet I do possess a large collection of professional testimony in favour of the inhaling treatment, and some of which I have already published. It is probable, however, that the larger part of the profession may not have paid any attention to it. Some, from theory only, never having made trial of inhaling, or even witnessed its effects, condemn it, as a hurtful irritant to the lungs. Some, on the other hand, also from theory, regard it as a feeble, doubtful, and very troublesome method. Others assure me that they have given a very fair trial to the inhalation of iodine and conium in consumption, according to my recommendation, with eventual failure, as with all other means of treatment of this lamentable disease, although at first much pleased with the remedy, and greatly encouraged with the hope of success. There are a few who pass a strong censure on the mode of receiving the medicated vapour by the tubes of the glass inhaler.

I shall endeavour to offer a running commentary on the several points which I have here stated.

I affirm that the inhalation of iodine and conium so far from irritating the air passages, either in tubercular phthisis or in chronic bronchitis, proves more or less soothing, provided it be used of proper strength, and that inflammatory action be not present—pleasingly soothing, I repeat, independently of its more remedial power, of which I shall have to speak. Notwithstanding the certain trouble of the process, the patient looks with pleasure to the hour of repeating it, from his confidence of receiving relief.

There may be some of the profession who, having witnessed very untoward effects from the internal administration of iodine, or occasionally, even from its excessive employment externally, dread its use even in the way of inhalation; and, as I now and then read in print, speak of the evils of this potent medicine so employed. But what remedy or treatment, either in the hands of the physician or of the surgeon, would not fall into discredit and even odium, if its merits were to be adjudged from the occasional accidents which arise, whether to be referred to the unfitness of the case, or particular symptoms temporarily existing; to error in the doses or mode of administration; or to idiosyncracy of constitution in the individual, which renders him an unfair example of the merits of the remedy employed? This position might be illustrated by a thousand examples. Some declare they would endure any pain, or continued loss of sleep, rather than swallow the least portion of opium, having experienced in their system, its distressing effects. Others, who from a few grains of blue pill have incurred a very severe salivation, would expect death itself to ensue from even a moderate use of calomel.

There are again others who call in question the efficacy of inhalation, and denounce it as a feeble and uncertain mode of practice, inferior to the routine administration of medicines by the stomach. Amidst such conflicting opinions and prejudices, who shall determine the truth?

"Utinam tam facile vera invenire possem, quam falsa convincere."—*Cicero.*

It might be supposed that time alone would serve to decide the real pretensions of any remedy introduced into general use; but in reality the influence of fashion extends even to medicines; as we have often seen that a favourite remedy, popular alike with the profession and the public, has, after no distant interval, been condemned, discarded, and forgotten. Yet it has again been the fate of several medicines so discarded to return into favour, and enjoy a fresh reign.

It is therefore extremely difficult to discover the truths of physic, and to establish on solid grounds the just claims to confidence which any particular medicine, or mode of treatment, may really possess. Preconceived opinion and prejudice too commonly stand in the place of reason and dispassionate inquiry, and oppose the advance of truth. The dangerous nature of consumptive disease, and its great fatality, should rather serve to stimulate our industry to discover some method of lessening the force of the scourge, than to be passive spectators of its dire results. The high authority of Laennec may probably have had some influence in confirming the general opinion of the incurable nature of phthisis pulmonalis. The distinguished author observes (Forbes' Translation, p. 305:)—"The observations contained in the Treatise of M. Bayle, as well as the remarks made in the present chapter on the development of tubercles, sufficiently prove the idea of the cure of consumption in its early stage to be perfectly illusive. Crude tubercles tend essentially to increase in size and to become soft. Nature and art may retard or even arrest their progress; but neither can reverse it. But while I admit the incurability of consumption in the early stages, I am convinced, from a great number of facts, that in some cases the disease is curable in the latter stages—that is, *after* the softening of the tubercles and the formation of an ulcerous excavation."

The attempt, therefore, to treat tubercular phthisis by any novel mode, with the expectation of success, is thought probably to wear the appearance not only of presumptuous boldness, but of the vain pretensions of quackery* itself. Far be it from me to speak in any light terms of this fearful disease, or to boast that I have certain means of cure at my command, in any case which may present itself.

I have now enjoyed no short term of medical life, and can well remember the results of different methods of practice in this disease in former years, without any success resulting. For a long period it was a favourite practice to put the patient on very slender diet, perhaps of milk, vegetables, and fruits, exclusively; and especially on the use of digitalis at the same time; on the theory of procuring a quiet state of the circulation, and preventing irritation and over-action of the lungs, by keeping

* I have occasionally met with observations of this nature applied to myself, with reference to inhalation; but they have always been either deficient in courtesy, or so stamped with scurrillity, and vulgarity of style, that they have never called for my serious notice, or more than my silent contempt. Written in the same spirit of low detraction, some letters have lately appeared in the Lancet, doing equal discredit to the head and heart of the writer, respecting the London Dispensary for the Diseases of the Lungs. The prosperity of the institution will be the best refutation of such puny attempts to injure either the Charity itself, or its Medical Officers.

the formation of the blood within the narrowest limits, as to its quantity; and abating its stimulating quality also, by lessening the density of its red particles.

Finding the constant failure of this and other modes of treatment, about twelve years ago I first made trial of the inhalation of iodine and conium, not having then heard of any experiment of the kind being made by others. I was gratified by a degree of success which I had never obtained from any other means; and I published some of my results in November, 1830. But great as is the importance which I attach to this one remedial method, I should be sorry to have it supposed for one moment that I would depend on it alone. On the contrary, I am fully aware how essential a matter it is to treat the whole constitution; such treatment being modified according to the circumstances of the individual case. As a general principle, I am an advocate for a very supporting plan of diet, and the use of corrective tonic medicine, combining with it the occasional careful administration of *alteratives.* Good air, the avoidance of vicissitudes of temperature, while, at the same time, a due ventilation is well maintained in all the apartments which the patient occupies, are points of great importance. It is not sufficient that we attempt to relieve the lungs from the irritation of tubercles at present existing; but we must endeavour to remove the tubercular *diathesis*, and counteract the tendency to fresh formation of tubercles. Hence it follows, also, that when a consumptive patient may have had the good fortune to be benefited by treatment to the extent of a tolerable recovery, it is incumbent upon him to lead a life of exceeding care afterwards, in regard to diet and regimen, clothing, place of residence, and in every material particular relating to health; in order that a relapse may be prevented.

It is a question of the highest interest to consider whether we may not, in contradiction of the gloomy declaration of Laennec, undertake the cure of the early stage of tubercles, with the hope of success? I have, in numerous instances, by means of inhalation, and *combined* treatment, succeeded in removing the early state of tubercular irritation, and which had been clearly manifested by the signs revealed by auscultation and percussion, by great elevation of the animal heat, and by the concurrent symptoms of cough, short breathing after quick exercise, frequency of pulse, hectic fever of greater or less amount, wasting of the body, and loss of strength.

As an example bearing exactly on this point, I will advert to the case which I published in the second edition of my work on Inhalation, &c., p. 66, I stated, in the observations on the case, p. 73, that, from the several indications, it was reasonable to believe that tubercles existed. The recovery of the patient under the treatment adopted was most satisfactory. He remained quite well for upwards of a year, and continued so, with a slight exception, for a much longer period, enjoying the sports of the field, and displaying great strength of body in his various exertions. He resided in a distant part of Scotland. But, presuming most imprudently on his state of recovery, he committed excesses at the table, with late hours at night, and carelessness of exposure in the day. Under these circumstances he was attacked most severely by the epidemic influenza, from which he never fairly recovered. He came under my observation about a year afterwards, when, upon examination, I found, on the upper part of the right lung, the clearest signs of the existence of a cavity. The pectoriloquism was most strongly marked. The left lung was also much diseased. Under a course of treatment he amended greatly; but again having neglected himself, and suffering from a fresh attack of influenza, he relapsed into a hopeless state, although he combated with his disease for a considerable length of time.

Is it not evident, from the indications by auscultation, as well as from the physical symptoms stated in the work referred to, that the right lung was tuberculated when I began the treatment in March, 1830? and that the remedies removed all irritation of the lung? For, according to his own cheerful letter, six months after, he described himself as strong and fat, and without cough. Whether absorption of tubercles had taken place, or a change in their condition been produced, or that the state of the lung was so altered as no longer to be irritated by the tubercles as foreign bodies, would be matter of theoretical speculation. It will, perhaps, be said that it was not a recovery, because he afterwards died from the disease: but he did enjoy a long season of health; and, had he led a prudent life, might probably have avoided the subsequent danger which he incurred.

The lady, whose case I relate at page 138 of the work before mentioned, is at the present time enjoying very comfortable health; and her lungs, as I had lately the opportunity of ascertaining, are quite free from all signs of disease. Nine years, therefore, have elapsed since the period when I first visited her, and found her in the state which I described at the time in the following words:—"She was very much emaciated, was so extremely weak, with such hollowness of cheeks, and such looks of sinking, that my first impression was that of distress from the apprehension that the case was beyond the reach of any medical treatment. The pulse ranged from one hundred and twenty to one hundred and thirty, and was occasionally more frequent; the cough was violent, and so peculiarly harassing at night, that the sleep was constantly disturbed. The expectoration was difficult, partly coloured with blood, the whole of a very puriform appearance, and in quantity about four ounces in the twenty-four hours. There were morning and evening accessions of hectic fever. The night perspirations were so profuse, as completely to saturate the sheets with moisture. She was so reduced in strength, that she required to be carried from the bed to the sofa in the adjoining room. At the upper part of the right lung there was pretty well-marked pectoriloquism and strong *gargouillement.*"

This quotation from my narrative of the case will be sufficient to show its great importance, and the value of the treatment. Fortunately for my exertions, this lady, distinguished in her character by every estimable quality, possessed that first of virtues in a patient—entire obedience. She did complete justice to all my recommendations, and was eventually rewarded with a return of health.

The patients, whose symptoms of tubercular phthisis, with the treatment, have been fully described in this Gazette, have not had any relapse, and are now enjoying excellent health, a period of rather more than five years having elapsed.

In the following case of a young lady, aged twenty-four, whose sister had died from consumption, the inhalation of iodine and conium rendered the most satisfactory relief and lasting benefit.

In the history which she gave me of her case, she stated that, in the year 1830, she had fallen into a very delicate state of health, in consequence of a chest complaint. She went abroad, in the hope of re-establishing her health; and which object was in a great measure effected by residing five months at Nice. Yet, living again in England, she experienced a relapse of her disorder in 1832; and such was the delicacy of her chest, that any slight exposure to a damp or cold atmosphere was almost certainly followed by pulmonary disturbance; her symptoms being, shiverings, succeeded by heat of skin and perspirations; cough and shortness of breathing; with a general soreness of the chest, and a sense of constriction, attended with debility and great depression of spirits.

Under such circumstances I was consulted; and, upon examination of the chest by auscultation and percussion, I had the clear evidence that the upper part of the right lung was much tuberculated; but on the left side the respiration was natural, with the exception of some slight râles. I adopted my usual plan of treatment, the particulars of which I will not detail. The result was most satisfactory. My patient described that she "felt from the inhaling a soothing and healing effect; soreness and pain were soon removed; and she became sensible of a freedom and expansion of the chest to which she had long been a stranger. The relief which she experienced gave her the idea of long-closed valves being re-opened and set free." After a few weeks, all the troublesome symptoms passed away. By pursuing a careful system of management, medical and dietetic, and paying strict regard to regimen, this young lady regained her health; and, I have every reason to believe, has continued well.

In the beginning of June, last year, I was consulted by a gentleman, aged thirty-five, long an invalid from pulmonary disease. He had resided many years in the West Indies, from whence he came in what he felt to be a hopeless state of suffering. I found him in bed, too weak to leave it. There was an assemblage of the most urgent symptoms; a frequent and very weak pulse; the animal heat 99;* urgent cough, with difficult expectoration of an offensive puriform sputum,† occasionally coloured with blood; the chest much oppressed, and the breathing quick and uneasy on the least exertion, with occasional pain in the sternum and intercostal muscles: he had hectic fever, and night perspirations, which were not only profuse, but of a peculiarly faint and disagreeable odour. Sleep slight and unrefreshing; without appetite, and the functions of the liver unhealthy; much reduced in flesh, and having coldness, and considerable œdema of the ankles and feet. At the upper part of the right lung there was strongly marked pectoriloquism to a great extent, with *gargouillement*, indicating extensive cavity; the respiration almost wholly bronchial, with sibilant râles. On the left side the perspiration was imperfect in some parts, in others puerile, and there were occasional râles, but without pectoriloquism. Percussion confirmed the signs of tubercular obstruction in each lung, but especially in the right, which was scarcely in the least degree capable of its functions. The right side of the chest was flatter than the left, and rose but little in a forced inspiration. His mind was in a state of the utmost despondency; and, contrary to that buoyancy of hope which prevails in acute phthisis especially, but often also in chronic, he had a fixed persuasion that he should not recover.‡

* Without exception, I have always found a high degree of the animal heat in tubercular phthisis; showing, as I conceive, a specific irritation present, allied to inflammatory action, yet different from true inflammation.

† The nature and quantity of the sputum is highly instructive, as regards the disease in its seat, stage, and intensity.

‡ Apart from the great consideration that we are all in the hands of a Superior Power, and cannot know when we may be called away, we do see, as medical observers, the extraordinary difference in the strength of the vital principle, if I may so express myself, in different individuals. Some yield their life to apparently slight assaults of disease; while others recover under circumstances seemingly the most hopeless. Such curious results should, I grant, render the physician very modest in the boast of his art, and make him study nature the more at-

The physician who had been in close attendance for six weeks, apprehended a fatal termination of the case. The limits to which I think it necessary to confine myself on the present occasion, will not allow of minute details in my account of the treatment. The patient inhaled the mixture of iodine and conium regularly three times a day, at first for ten minutes, afterwards gradually increased to twenty; small blisters were applied to the chest from time to time: the lotion of tannin infusion, with acetic acid and eau de Cologne, was applied night and morning to the skin, followed by the use of the flesh-brush. Internally, pills composed of pilula hydrarg. camphor, and c. colocynth extract, were given at night, occasionally, followed by a morning aperient draught; a strong infusion of the cortical part of sarsaparilla, with alkali and gentian, was used twice in the day; and, to procure comfortable sleep at night, he took a soothing morphine syrup, acidulated with diluted sulphuric acid. The plan of diet was changed to one highly nutritious; and such was the languor and debility, that wine, the best port and sherry, was allowed with more than usual freedom. He usually took three or four glasses in the course of the day, in addition to a pint of sound draught porter, not only without disagreement, but with every sense of benefit. He had sometimes alarming attacks of exhaustion, at the commencement of my attendance; and he had indeed said that he was "dying from inanition." After a few weeks, iron and quinine were administered in conjunction, instead of the other medicines.

So beneficial was the whole treatment, that, in rather more than a fortnight, the specific symptoms were most materially relieved, and the strength and spirits were greatly regained. The night perspirations had nearly ceased. As a proof of the amendment of the lungs, he could, in the beginning of July, walk two or three miles, at a quick pace, without resting. He improved progressively. In September he travelled. I saw him again at the end of November, and found a remarkable diminution in the extent of the pectoriloquism, with an evident amelioration in the condition of each lung. The râles had ceased, and by auscultation* there was satisfactory evidence of a very improved respiration. The expectoration continued, but was much lessened in quantity, and almost free from its former offensive odour. It appeared to me that the tubercular cavity was in a favourable progress of healing; and certainly the whole aspect of the patient was promising for a fair recovery; for, to regain perfect health could not be expected, when so much disorganization of lungs had been produced, existing in conjunction with an unhealthy liver. In my early attendance, I was struck with his cadaverous and dark complexion; and this unfavourable omen disappeared in a few weeks. He related to me that at various periods he had experienced slight hæmoptysis. Under my own observation, in about seven weeks from the commencement of my attendance, he used a warm bath, not exceeding 96° in temperature, and was remarkably refreshed by it; but on the same evening hæmoptysis occurred; half an ounce of pure red blood issued with a cough. This hæmorrhage I attributed to the excitement which the circulation had received from the bath. It is satisfactory to state that on no occasion did the inhalation give rise to this accident;* and he always felt more or less of sensible relief from it. This and other treatment was continued, with occasional intermissions, during five months, when he had regained flesh and strength; could walk six or eight miles in the day, and felt himself sufficiently recovered to return to the West Indies, for a period necessary to arrange his affairs.†

It has been my purpose to show that, in all the stages of tubercular phthisis, it is our duty to enter upon a more or less active systematic plan of treatment, and that we do so with less discouragement of success than Laennec and others have taught. Yet no one can be more aware than myself of the danger of this disease, and its too frequent mortality under every care and exertion to ward off such a termination. I always wish, therefore, to be understood as speaking of the cure of consumption in a very guarded and qualified sense. I can truly declare that I have had the gratification of very often succeeding in bad cases, where, according to all my earlier professional experience with other treatment, I should have failed.

I am not only convinced of the excellence of the remedy—the inhalation of iodine and conium—but also of its superiority, as a curative agent, over chlorine and creosote; yet, I must again observe, "let it not be imagined that I limit myself to this treatment, which I would rather speak of as a most valuable auxiliary, tentively, that he may have acquaintance with all her ways.

* I may take this opportunity of mentioning that I derived considerable assistance in auscultation from placing a sheet of writing paper over the chest, and listening through this medium.

* I have at present under my care a lady, who, in the course of her illness, before I was consulted, had frequent attacks of hæmoptysis, which have not been reproduced by inhalation. In case of hæmoptysis, whatever the exciting cause might be, I should suspend the inhaling till the disappearance of any blood. This patient has tubercles and a cavity. Her amendment has exceeded my most sanguine expectations.

† This gentleman was staying, during my attendance, at the house of Mr. King, surgeon, Portland Terrace, St. John's Wood, who can bear full testimony to my account of the case. To his kind care and attention the patient was much indebted.

than as the sole means of benefit." It is incumbent on us to look comprehensively to the state of all the constitutional functions; to attack the tubercular diathesis; to control to the utmost of our power the nutritive functions, from the first digestion of the food in the stomach to the succeeding processes of chylification, lacteal* absorption, assimilation, and sanguification; and to effect a change in the whole mass of blood. With this large view, we are required to combine, with the strictly medical treatment, a precise plan of diet and regimen; of exercise in the open air,† in a manner adapted to the patient's state, in the favourable season of the year; and, in weather precluding out-doors exposure, to direct the arrangement of a medium rather than a high temperature of the apartments, always paying great attention to their fit ventilation. And shall the execution of such pathological and practical views be termed empirical, and without claim to respect?

I know that, in a very large proportion of cases of consumption, there must be eventual disappointment to our hopes; but of this fact I am certain, that it is generally in our power to palliate all the sufferings; to afford very great relief; and to prolong life. It unfortunately happens, and this it has been greatly my fate to experience, that the disease has arrived at its last stage, and when extreme disorganization of the lungs has taken place, before an active plan of treatment has been adopted; so that the opportunity of rendering material benefit has been irrevocably lost. The disease does, indeed, often effect its march most insidiously; and danger is present almost before the sense of illness is felt, or at least acknowledged.

I have, on different occasions, entered my protest against sending the unfortunate patient, as is so commonly done, in a confirmed state of the disease to a warm climate; trusting for benefit almost, perhaps wholly, to its influence. This usually proves a journey to a foreign grave. Rather let us, however late the attempt, and with however poor a prospect of success, enter upon the attentive treatment of the case; and of which, according to my views and experience, inhalation will prove a most valuable part. Here let me again ask, whence the objection? All other means in which medical confidence may be placed may be equally employed without any deduction of benefit from the use of inhalation, which, in theory, from its being a direct application of a potent remedy to the seat of disease, so much recommends itself; and from which I am confident more or less of benefit will always arise. I am quite sure that every practitioner, in the treatment of a consumptive case, feels the anxious and difficult ground on which he treads, and may be well pleased to add to his expedients any one remedy uniting in itself efficacy and safety. I will not attempt to say what proportion there may be, but I regret to admit it must be large, of the cases of true tubercular phthisis which eventually resist all power of medical skill. Yet, is this a reason for supineness and indifference in the mind of a physician? As in all difficulties, let us increase our exertions in proportion to the obstacles which we meet. It is not the reproach of good remedies that they cannot heal immedicable wounds!

I cannot, I think, too often repeat, that while I claim for the inhalation so great a regard, I consider it to be only one part of the treatment required. The additional constitutional means embrace a very wide consideration. The local external treatment of the chest by proper means of counter-irritation, and by lotions and frictions, is a very important part of management.

I have never seen, from an active remedy, so large a proportion of benefit with so small a proportion of disagreement and inconvenience, as from the inhalation of iodine and conium. The method also is to be considered: and I may here remark, that many excellent remedies have fallen into odium and neglect, at different periods, from the error or abuse of their application. I am careful that all the ingredients which enter into the composition of the inhaling mixture are perfectly pure.* I recommend the following formula:

℞. Iodinii puri,

Iodid. Potassii $\overline{aa}$ gr. vi.,

Aquæ distillat. ℥v. ʒvi.,

Alcoholis ʒii.

M. fiat mistura, in inhalationem adhibenda.

I now always prefer to add the conium at the time of mixing the iodine solution with the water; and it should be a *saturated* tincture, prepared with the most genuine dried leaves. In the commencement of the treatment I advise very small proportions of the iodine mixture; for example, only from half a drachm to a drachm for an inhaling of eight or ten minutes, to be repeated two or three times a day. Of the soothing tincture, I direct half a drachm—which I usually find sufficient; but it may be increased if the cough be very troublesome. I soon augment the quantity of the iodine, and progressively from ʒj. to ʒiv.; but also, then

* The idea of an unhealthy action in the mouths of the lacteals, was first submitted to my consideration by Dr. Sherriffs. To the loss of their peculiar sense of discernment between organizable and disorganizable matter, and the consequent indiscriminate absorption, he attributes all tubercular disease.

† The use of that most valuable apparatus, the Respirator, in certain states of the atmosphere, is very important.

* The various medicines to be used for inhalation may be obtained in perfect purity from Mr. Garden, Oxford street. Many other chemists, without doubt, also have the articles in question correctly prepared.

prolonging the time of inhaling, I divide the iodine dose, putting two-thirds at first, and the rest after the expiration of seven or eight minutes. If the temperature of the water be measured by the thermometer, it should be 120° Fahr. as being the most favourable for volatilizing the active principles of the iodine and conium, mixed with some watery vapour; but the approximation will be sufficient, if equal parts of boiling and cold water be used; with which the inhaler is not to be quite half filled. Invariably, however, care should be taken to prepare the bottle for this heat of water, by first washing it out with some tepid water.

During the process, the inhaler should be kept immersed in a jug containing water of rather higher temperature than 120°.

It is of the utmost importance that the strength of the inhaling mixture should be considered in relation to the particular case;* the feelings of the patient will be a great guidance. He should have the sense of relief, and not of inconvenient irritation, produced. The cough arising occasionally during the process is not an objection; but if it be more irritable afterwards, it shows that it has been used too strong. There is a certain stage of the tubercular disease, when over-excitement, from employing the iodine in too great quantity, might hurry on the softening process too quickly. It is here that the treatment demands the greatest judgment. In every case one of the following events may be expected to happen: either that the tubercular irritation will be arrested and gradually removed, be arrested and suspended, but not cured; or pass on to the softening process, which terminates in the production of an excavation. In all these different states of disease I advise the inhaling treatment to be employed.

I have formerly mentioned that at the period when I was preparing my first edition for publication, in the year 1830, Dr. Murray, of Belfast, in a treatise on Animal Heat, recommended the introduction of iodine to the lungs, diffused through warm aqueous vapour into the apartment; and hence it follows that without any communication—for I had not the pleasure of his acquaintance—we had thought of the same remedy; but I am well persuaded that the only certain and exact mode of employing it is by direct inhalation, so that the dose is defined; whereas, in the other mode, many circumstances must interfere with its regularity. Also I find the remedy infinitely improved by the addition of the conium.

In the Medical Gazette for April 6, 1839, we find a paper by Dr. Corrigan, of Dublin, recommending, as a mode of inhalation, the impregnation of the atmosphere of the apartment with iodine vapour, by a mode different from that of Dr. Murray; but the principle is of course essentially the same. I am gratified with the favourable testimony which this physician bears to the remedial influence of iodine vapour in phthisis pulmonalis; but I am not disposed to choose his method of employing it. The volatility of the iodine would cause the vapour to ascend to the highest parts of the apartment;* it would attach itself to the linen furniture, and must, of necessity, find its way to the lungs of the patient in a very uncertain degree of strength. The objections of Dr. Corrigan and others to the direct method of inhaling which I recommend, is without foundation. It does not, as they state, cause irritation to the larynx and air-passages; but, on the contrary, its influence, if used of the proper strength, is soothing and agreeable. The addition of the conium divests the iodine of that irritating effect which would arise from its penetrating acrid qualities when used *per se.*

But we have a higher purpose to fulfil than the mere study of soothing the mucous membrane of the air passages. The modus operandi of remedies is a question of secondary importance to their real effect, and may lead to endless controversy. It is my belief that this direct and very accurate mode of applying this powerful medicine, iodine with conium, induces a new action in the vessels and nerves of the lungs, which is calculated to supersede the diseased action. I also assign much effect to the stimulation of the absorbents, and have been lead to believe that tubercles have in this manner been actually removed.

As the *mode* of conducting the inhaling process is of such sovereign importance, I shall be excused, I trust, if I enter at some length into this part of the subject.

Dr. Harwood of Hastings has lately published an account of the benefits to be derived from inhalation, in which he recommends his newly invented tin inhaler, stating, as the ground of preference over the glass inhaler commonly in use, that it is used with such perfect facility as to prevent fatigue; and, what I must admit, that it is not liable to be broken. I have carefully examined the action of this inhaler, and must take the liberty of offering the following criticism. The ingress tube not dipping into the water, the fresh atmospherical air which enters when the patient inhales, cannot be more than slightly impregnated; and

* In acute phthisis, the inhaling doses should be very weak. No remedy with which I am acquainted exerts so much influence over the hectic fever, used in the intervals, as the inhalation in question.

* Nor is the great *waste* of the iodine a slight objection to this method. The author states that in the use of the apparatus, "about six drachms of the tincture of iodine will be evaporated in an hour;" and when he has it at work, as he says, "from eight to twelve hours out of the twenty-four," it would form no small item of expense, employed in a charitable institution!

any one making a comparative trial with the glass inhaler, is at once made sensible of the great difference in the strength of the inhalation, from equal quantities of the ingredients used. Indeed this great defect in the construction of the apparatus will apply to any kind of medication of the water. As regards the use of iodine and chlorine, the objection of the metallic nature of this inhaler is a fatal one. I find that an action immediately takes place between these peculiar medicines and the tin, which weakens their properties very considerably. With the requisite alterations in the ingress tube, this will be rendered a very useful inhaler, when it is desired to employ herbs or gums; water of a higher temperature than 120° being mixed with such articles to bring out their volatile principles; although the patient should wait the reduction of such temperature to the proper degree, or he would be injured by the direct application of so much heat to the sensible surface of the air passages.

I have not been able to persuade myself of the advantage of the crescent-shaped mouth-piece in Dr. Harwood's inhaler, over the flattened one of the glass tube.

With a well constructed glass inhaler I find all the satisfaction I can desire. The bottle should be large, and the tubes capacious. The one issuing from the bottle should be upright, passing off in a gradual slight curve, so that the vapour should not be much cooled in the course of its progress; the ingress tube should dip very near to the bottom of the bottle, that all the air so introduced may receive impregnation. The patient must be desired to inhale by using at the same time suction and a pretty full inspiration, then to drop the under lip from the mouth-piece and make a free expiration; so conducting the process by pausing, and, if he like, little suspensions, in order that he may not experience any of the fatigue, which would certainly happen if breathing quickly, or using an inhaler with small tubes, or with too much water in the bottle.

A little practice also improves the power of the respiratory muscles, so that any little difficulty, which may by possibility be felt at the first by a very delicate and nervous individual, is soon overcome. When care is used to prepare the inhaler properly, the accident of fracture is easily guarded against. With respect to the influence of other medicines used in this way of inhalation, I beg to refer the reader, who is curious on the subject, to the second edition of my Cases in illustration.

Thus I hope to have made a useful addition to my former statements of the importance of the inhalation of iodine and conium in tubercular phthisis, as constituting one most valuable part of the systematic plan of treatment which I recommend; and if I should ever have appeared to speak of it as the sole and exclusive remedy to be employed, and to be used empirically, I have not done justice to myself, and to my enlarged views of the pathology and treatment of consumption.

Did I not feel the necessity of restricting myself, in the discussion of my subject in this letter, I could much increase the evidence of the success of the treatment which I wish to advocate, by the relation of numerous cases of great amendment, or recovery, from tubercular phthisis in its different stages. I might also bring forward instances of relief and cure of various conditions of tracheal and bronchial disease, effected by means of inhalation and combined treatment.

I will, however, now conclude by observing, in the language of the poet to the enemy of the inhaling practice, if such there should continue to be,

> "Si quid novisti rectius istis,

> Candidus imperti: si non, his utere mecum."

> *Horat.*

In the treatment of tubercular phthisis, so much the most mortal of all diseases, no one need apprehend that he shall add unnecessarily to his means of giving relief.—*Lond. Med. Gaz.*

---

*Tasteless Form of Ipecacuan.*—When it is desirable to administer ipecacuan to refractory children, or to persons to whom the ipecac. wine is particularly odious, as is often the case, the following form will be found to answer:—

*Bruised root of ipecac.*, ½℥.

*Boiling water*, enough to make 1½℥.

*Lemon syrup*, ½℥. A twelfth part every third hour.

*Dr. Osborne, Dub. Jour.*

---

*Dr. Giboin's experience in the use of Soot in certain affections of the Bladder.*—Out of six cases of chronic inflammation of the bladder, four were cured by means of the following injection; the remaining two, which had ulceration in the fundus of the bladder, died. The injections were newly prepared and repeated thrice a day. Two ounces of chimney soot, as perfectly as possible freed from foreign bodies, were allowed to boil for six minutes in a pound of water, and the decoction filtered through paper. The pain was relieved immediately after the first injection. Dr. Giboin administered the soot internally in two cases of catarrh of the bladder which were almost despaired of, and with the best results. It was given in the form of pills, beginning with 4 grains daily, and increasing it soon to 16, 20, and 30 grains. These pills were prepared by boiling the finest soot for some minutes, filtering it through paper, evaporating the filtered fluid in a porcelain bason over a slow fire or sand bath to the consistence of an extract which is made up into pills,—*London Medical Gazette, from Medizin. Jarbuch des k.k. Osterreichischen Staates.*

